# Effectiveness and Acceptability of Conversational Agents for Smoking Cessation: A Systematic Review and Meta-analysis

**DOI:** 10.1093/ntr/ntac281

**Published:** 2022-12-12

**Authors:** Linwei He, Divyaa Balaji, Reinout W Wiers, Marjolijn L Antheunis, Emiel Krahmer

**Affiliations:** Department of Communication and Cognition, Tilburg School of Humanities and Digital Sciences, Tilburg University, Tilburg, The Netherlands; Amsterdam School for Communication Research, University of Amsterdam, Amsterdam, The Netherlands; Addiction Development and Psychopathology (ADAPT)-Lab, Department of Psychology, University of Amsterdam, Amsterdam, The Netherlands; Centre for Urban Mental Health, University of Amsterdam, Amsterdam, The Netherlands; Department of Communication and Cognition, Tilburg School of Humanities and Digital Sciences, Tilburg University, Tilburg, The Netherlands; Department of Communication and Cognition, Tilburg School of Humanities and Digital Sciences, Tilburg University, Tilburg, The Netherlands

## Abstract

**Introduction:**

Conversational agents (CAs; computer programs that use artificial intelligence to simulate a conversation with users through natural language) have evolved considerably in recent years to support healthcare by providing autonomous, interactive, and accessible services, making them potentially useful for supporting smoking cessation. We performed a systematic review and meta-analysis to provide an overarching evaluation of their effectiveness and acceptability to inform future development and adoption.

**Aims and Methods:**

PsycInfo, Web of Science, ACM Digital Library, IEEE Xplore, Medline, EMBASE, Communication and Mass Media Complete, and CINAHL Complete were searched for studies examining the use of CAs for smoking cessation. Data from eligible studies were extracted and used for random-effects meta-analyses.

**Results:**

The search yielded 1245 publications with 13 studies eligible for systematic review (total *N* = 8236) and six studies for random-effects meta-analyses. All studies reported positive effects on cessation-related outcomes. A meta-analysis with randomized controlled trials reporting on abstinence yielded a sample-weighted odds ratio of 1.66 (95% CI = 1.33% to 2.07%, *p* < .001), favoring CAs over comparison groups. A narrative synthesis of all included studies showed overall high acceptability, while some barriers were identified from user feedback. Overall, included studies were diverse in design with mixed quality, and evidence of publication bias was identified. A lack of theoretical foundations was noted, as well as a clear need for relational communication in future designs.

**Conclusions:**

The effectiveness and acceptability of CAs for smoking cessation are promising. However, standardization of reporting and designing of the agents is warranted for a more comprehensive evaluation.

**Implications:**

This is the first systematic review to provide insight into the use of CAs to support smoking cessation. Our findings demonstrated initial promise in the effectiveness and user acceptability of these agents. We also identified a lack of theoretical and methodological limitations to improve future study design and intervention delivery.

## Introduction

Cigarette smoking is one of the major causes of preventable death and premature diseases, contributing to more than 6 million deaths per year worldwide.^1^ Recent surveys indicate that almost 70% of smokers have the intention to quit smoking, and over half of them have made a quit attempt in the past year; however, the actual cessation rate remains low.^2,3^ Research shows that aided quit attempts are more likely to succeed than unaided quit attempts,^4^ and developing ­effective cessation interventions has been a public health priority. There is good evidence for the effectiveness of therapist-delivered interventions, such as brief advice, individual and group counseling, and telephone counseling.^4^ While such support provided by healthcare professionals is effective, the use of it remains low. A large-scale survey among smokers in a number of western countries shows that less than 20% of smokers have made use of cessation services during a quit attempt.^5^ The major reasons included the need for on-site visits and lengthy waiting times due to staff shortages.^6^ To combat these challenges, innovative digital tools such as conversational agents (CAs) have become increasingly popular in the healthcare domain.

CAs are computer programs that use artificial intelligence to simulate a conversation with users through natural language.^7^ Examples of CAs range from digital assistants such as Siri (Apple) and Alexa (Amazon) to customer service agents available on commercial websites.^8,9^ Automated synchronous text-messaging systems are also considered as a form of CA as they allow two-way communication between a human user and the computer system. More recently, CAs are being used to assist healthcare services because they are always accessible, can engage users in human-like conversations, and ­provide personalized contents to multiple users simultaneously. Evidence has begun to accumulate around the benefits of CAs in diverse fields including disease diagnoses, medication monitoring, mental health, and risk communication during the recent COVID-19 pandemic.^11–14^ However, to the best of our knowledge, there is no systematic investigation of the effectiveness of CAs for smoking cessation. Additionally, the accessibility of CAs may appeal to certain hard-to-reach groups and populations that face physical and time constraint barriers (eg, lack of resources and lengthy waiting time) to accessing traditional interventions, such as at-risk youth and individuals with low socioeconomic status.^10^ To extend access to rural, hard-to-reach individuals, it is essential to gain insights into user experience and design CAs that meet their needs and preferences.

Given the potential and the infancy of the use of CAs for smoking cessation, there is a clear need to systematically summarize the available evidence regarding the use of these agents to inform future development and adoption of them. As the effectiveness and user experience are intricately related,^15^ the aim of this systematic review and meta-analysis is to evaluate both the effectiveness and acceptability of CAs for smoking cessation. We aim to identify (1) characteristics, functions, and core conversational features of the CAs; (2) theoretical and technical foundations of the CAs; (3) user experience and needs; and (4) limitations and areas for future work. The results of this review will provide insights into the future design and implementation of CAs in smoking cessation interventions.

## Methods

This systematic review was conducted in accordance with the protocol (CRD42022313055) registered at the International Prospective Register of Systematic Reviews (PROSPERO) and is reported following the PRISMA (Preferred Reporting Items for Systematic Reviews and Meta-Analyses) guidelines.^16^

### Eligibility Criteria

Studies were included if they were published in English and if they: (1) had been peer-reviewed; (2) reported on the use of a CA or synonymous system (eg, automated synchronous text-messaging systems) that allowed autonomous two-way interaction without support from a human; (3) addressed smoking cessation or relapse prevention; and (4) reported outcomes evaluated from the direct end-users. Studies were excluded if the communication was one-way where the CA messages could not be responded to by the user (eg, reminders and pop-up notifications). Studies that only discussed the design or the development of the agents and reported no evaluation or the evaluation was not from end-users (eg, protocols and cost-effectiveness studies) were also excluded.

Considering the infancy of this field, we did not apply any restriction regarding the year of publication, population groups, or geographical locations to provide a comprehensive overview of the evolution of CAs for smoking cessation. Study design was not considered as a key restriction in this review. We included both randomized controlled trials (RCTs) and non-randomized studies (eg, before-and-after designs), observational studies, and qualitative studies.

### Search Strategy

A systematic search of peer-reviewed literature was performed using the following databases: PsycInfo, Web of Science, ACM Digital Library, IEEE Xplore, Medline, EMBASE, Communication and Mass Media Complete, and CINAHL Complete, with no restriction on publication date. Reference lists of included studies were searched to identify additional relevant literature. Two sets of search terms were used for the literature search. The first set addressed CAs and included synonyms such as “dialogue system”, “digital agent”, and “virtual coach”. The second set addressed smoking cessation and included keywords such as “tobacco control”, “smok* reduction”, and “smok* abstinence”. Full search strategies can be found in Supplementary File 1. Last searches were completed on March 1, 2022.

### Study Selection

**Figure 1.**
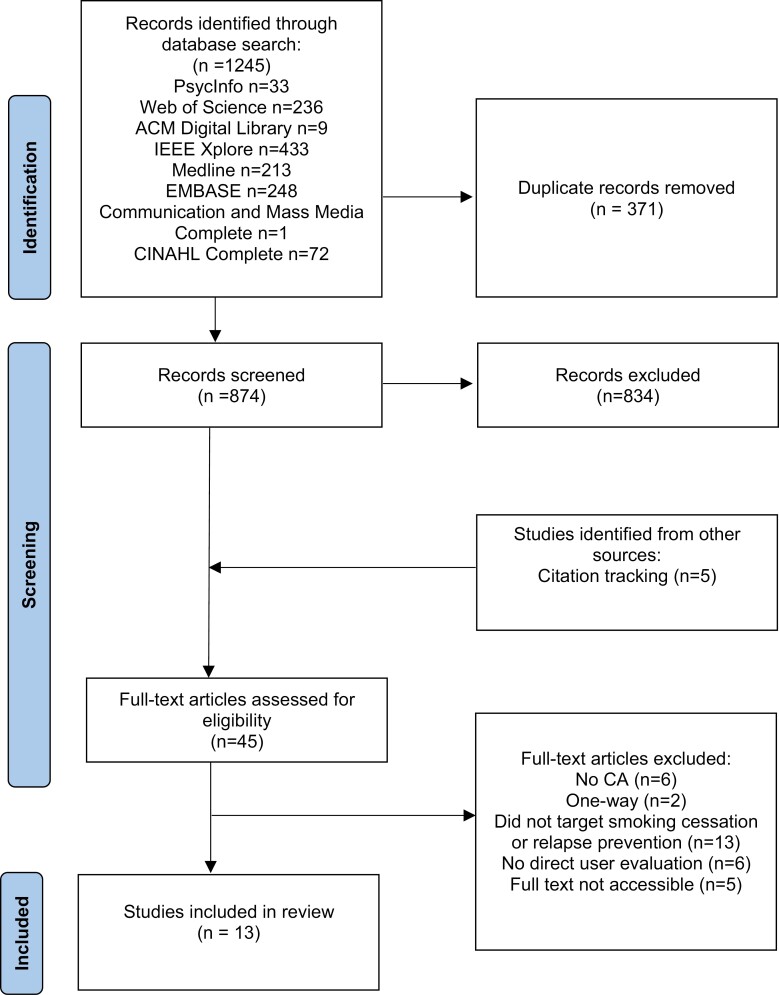
PRISMA chart of the selection process.

One thousand two hundred and forty-five records were identified from database searches and were imported to the review software Rayyan.^17^ After removing duplicates, 874 studies were screened based on title and abstract. A pilot screening (*k* = 90) was conducted independently by two reviewers (LH and DB). With substantial agreement (Cohen’s *k* = 0.71) achieved, one reviewer (LH) carried out the remaining abstract screening. Full texts of studies considered as potentially eligible were then screened by two reviewers (LH and DB) using the software EndNote, and any discrepancies were resolved by group discussion. Finally, reference lists of included studies were also searched and screened, resulting in 13 studies included in the present review with a substantial inter-coder agreement (Cohen’s *k* = 0.71). See Figure 1 for the detailed selection process.

### Data Extraction and Risk-of-Bias Assessment

Included studies were coded to capture information regarding the study characteristics (eg, study design, sample type and size, demographics), the CA system (eg, modality, initiator, artificial intelligence techniques, communication ­channel), the intervention characteristics (eg, theoretical framework, ­comparator, intervention duration, and interaction frequency), and the outcomes (smoking-related outcomes and user experience outcomes). A standardized Excel form was devised by the author team to record the data. One author (LH) extracted the data from all included papers.

Risk-of-bias assessment of each study was conducted to ascertain the validity and reliability of the study methods and findings to inform the results of this review. The Cochrane Collaboration Risk of Bias tool^18^ was used to appraise the quality of RCTs and the Risk of Bias in Non-randomized Studies of Interventions (ROBINS-I) for non-randomized interventions.^19^

### Data Synthesis and Analysis

A narrative synthesis was conducted to summarize the results of all the included studies and describe the effectiveness and user experience of current CAs for smoking cessation. Additionally, a meta-analysis was conducted with six RCTs that reported on abstinence (both biochemically verified and self-reported) to create an overall effect size. Abstinence rates were extracted and presented as odds ratios. The majority of studies reported abstinence at multiple endpoints (eg, at 1-month and 3-month follow-up), and we included outcomes measured at the longest endpoint unless a primary outcome was specified by the authors. A random effects model was adopted for the meta-analysis given the variation in the study designs and intervention content.^20^ Statistical heterogeneity was assessed using the *I*^2^ statistic with an *I*^2^ larger than 50% indicating significant heterogeneity and subgroup analysis is then warranted.^21^ Visual inspection of the funnel plot was used to assess publication bias. Additional sensitivity analyses were carried out excluding trials providing only self-reported abstinence and with a high risk of bias. All the statistical analyses were conducted using the Cochrane software ReviewManager (RevMan).^22^

## Results

### Study Characteristics

The key characteristics of the included studies are summarized in Table 1 and more details can be found in Supplementary File 2. The 13 included studies were conducted between 2012 and 2021 in eight countries including the United States,^23–26^ the United Kingdom,^27^ Canada,^28^ Japan,^29–31^ Hong Kong,^32^ Vietnam,^33^ Cambodia,^34^ and Switzerland.^35^ There is considerable variation in study design, CA characteristics, and intervention design across the studies. Most studies used mixed methods combining quantitative (eg, experiments) and qualitative (eg, interviews) approaches, and the majority reported on both cessation outcomes and user experience outcomes.^23–29,32–34^ Seven RCTs were identified, and the six remaining included studies utilized either pre-post or ­post-designs. Among the RCTs, the most common comparator was same application or devices as the experimental group but without the active CA element^27,30,32,34^; other comparators included guidebook^25^ and message with low intensity for data collection purposes only.^33^ Finally, one RCT comparing the CA with an extra active element (ie. CA plus peer mentoring)^26^ was also included for insights on user experience. Regarding the wide variation in study design, most studies did not mention prespecified or registered study plans (except for^26^ and ^27^).

**Table 1.** Characteristics of Included Studies

Authors	Country	Study design	Total *N*	Sociodemographic	Control	Total intervention duration	Interaction frequency	System platform	Initiator	Primary cessation-related outcome
Abdullah et al.^23^	United States	Pre-post	6	Male (66.7%), mean age 56.3 years, white (50%), at least some college education (67%), unemployed (83%)	NA	14 days	Daily	Tablet: App	System	Self-report 7-day abstinence at post intervention
Abroms et al.^25^	United States	RCT	503	Male (34.4%), mean age 35.7 years, white (78.5%), at least some college education (78.1%)	Guidebook	3 months	Daily, gradually decreased to weekly	Phone: SMS	Both	Repeated point prevalence abstinence, saliva verified
Abroms et al.^24^	United States	Pre-post	23	Male (56.5%), mean age 20.9 years, white (56.5%)	NA	4 weeks	Daily, gradually decreased to weekly	Phone: SMS	Both	Self-report abstinence at post intervention
Almusharraf et al.^28^	Canada	Pre-post	121	Male (48.4%), mean age 35.2 years, paid employment (67.2%)	NA	21.3 minutes	Once	Website	System	Self-reported perceived quitting benefits
Bui et al.^34^	Cambodia	RCT	50	Male (100%), mean age 44.2 years, years of formal education 8.5.	App without the CA component	8 weeks	Daily	Phone: App	System	7-day abstinence at 2-month follow-up, CO verified
Calvaresi et al.^35^	Switzerland	Pre-post	270	Male (27%)	NA	NI	NI	Phone: App	system	Self-reported abstinence at 3-month follow-up
Jiang et al.^33^	Vietnam	RCT	100	Male (98%), mean age 38.9 years, at least high school education (77%)	Assessment message	6 weeks	Daily	Phone: SMS	Both	7-day abstinence at 12-week follow-up, CO verified
Kato et al.^31^	Japan	Pre-post	177	Male (63.3%), mean age 44.6 years	NA	24 weeks	Daily	Phone: App	Both	Continuous abstinence 9–12 weeks, salivary verified
Masaki et al.^29^	Japan	Pre-post	51	Male (71%), mean age 43.3 years	NA	24 weeks	Daily	Phone: App	Both	Continuous abstinence 9–24 weeks, CO verified
Masaki et al.^30^	Japan	RCT	490	Male (75%), mean age 46 years	App without the CA element	24 weeks	Daily	Phone: App	Both	Continuous abstinence 9–24 weeks, CO verified
Perski et al.^27^	United Kingdom	RCT	6111	NI	App without the CA element	1 month	Daily	Phone: App	Both	Self-report abstinence at post intervention
Wang et al.^32^	Hong Kong	RCT	134	Male (75%), mean age 46 years	Educational information via phone	2 months	Daily	Phone: App	Both	Self-report 7-day abstinence at post intervention
White et al.^26^	United States	RCT	200	Male (22.5%), median age 45 years, predominantly non-Hispanic white (74.5%)	Extra peer mentoring	47–57 days	Daily	Phone: SMS	Both	7-day abstinence at 3-month follow-up, salivary verified

CA = conversational agent; RCT = randomized controlled trial; NI = no information; NA = not applicable.

The 13 studies represented a total of 8236 participants who were recruited in both clinical settings^23,29,30,33,34^ and nonclinical settings.^24–28,31,32^ Study sample sizes ranged from 6^23^ to 6111.^27^ For nine of the studies, the mean age of participants fell into the 35–64 years range. Two studies recruited younger participants with a mean age between 20 and 34 years. Two further studies did not provide information on this. For studies that provided socio-demographic information of the participants, most of their samples were balanced in terms of gender, with predominantly white participants with at least some college education. Most of the studies provided information regarding participants’ baseline smoking behavior (except^32,35^), and the mean number of daily cigarette consumption was between 11 and 20. Five studies assessed participants’ nicotine dependence level (also referred to as tobacco use disorder, according to the Diagnostic and Statistical Manual of Mental Disorders, Fifth Edition [DSM-5]) mainly using FTND (the Fagerström Test for Nicotine Dependence^36^) and TDS (the Tobacco Dependence Screener^37^), and the majority were moderately to highly nicotine-dependent smokers.^24,25,29–31^

### Risk of Bias

Of the 13 studies included, two were judged as having a low risk of bias,^25,33^ five having a moderate or unclear risk of bias,^27,29–31,34^ and five had a high risk of bias.^23,24,26,32,35^ The most common reason that contributed to a high risk of bias was possible deviations from the intended intervention, which included unequal amount of intervention received or adhered to by the participants. For example, the time that participants spent on interacting with the CA varied and not all participants read all the texts from or sent messages to the CA, and the on-demand option was not used by all participants.^24,25,33^ Only having self-reported abstinence was another source that frequently contributed to a moderate or high risk of bias.^23,24,35^ See Supplementary File 3 for the detailed assessment for individual studies and a visual summary of the assessed risk of bias.

### CA Interventions

The 13 included studies represented 9 unique CA systems. Among them, six were CAs operating in a chatroom format^23,27–32,35^ and three were automated synchronous text-messaging systems.^24–26,33,34^ The CA systems operated on different platforms, with seven accessed on an app on the phone,^27,29–32,34,35^ one on a tablet,^23^ one integrated into a website,^28^ and four using SMS services.^24–26,33^ Only one CA was embodied (ie. with visual representations of the agent).^23^ The majority (7 out of 13) of the CAs took natural written language,^27–32,35^ and 4 took responses from participants using fixed keywords.^24–26,33^ The remaining two studies did not specify whether the CA processed natural language.^23,34^ A large proportion of studies (10 out of 13) did not provide description on the technical architecture of the CA systems, and those who did all used a rule-based infrastructure, and no natural language generation techniques were involved.^28,32,35^ Most of the CA interaction was individualized (ie, the agent interacted with individual users separately), and one study was in a group chat setting where the agent facilitated the interaction between multiple users.^32^ The majority of the systems allowed both the agent and the user to initiate the interaction^24–26,29–33^ while the rest allowed only the agent to start the conversation. Lastly, two studies^23,34^ provided participants with mobile devices for the intervention, and participants from the rest of the studies used their own devices.

The CAs primarily aimed at smoking cessation promotion, and the dialogs were designed with the implementation of a range of behavioral change theories, including Social Cognitive Theory,^24,25,33^ Transtheoretical Model,^33,34^ Motivational Interviewing,^28^ and Cognitive Behavioral Theory.^33^ The remaining six studies did not provide information on the theoretical bases of CA development.

The CA interventions varied considerably in duration and frequency, ranging from one-time interaction^28^ to daily interaction over 24 weeks.^29–31^ Multi-session interventions all involved daily interaction, with a few studies gradually decreasing the frequency to at least weekly.^24,25^ Six of the studies used the CAs as a stand-alone intervention, while the rest combined the CAs with other intervention components such as personalized web portals and emails^24,25^ and other non-interactive educational information in the app.^27,29–31,34^

### Effectiveness Evaluation

All 13 studies assessed smoking cessation-related outcomes but with great variation in the measures. Eight studies reported biochemically verified abstinence using either salivary cotinine or carbon monoxide readings^25,26,29–34^ while three studies included self-reported abstinence only.^24,27,35^ In addition, two studies did not assess abstinence but reported on other cessation-related outcomes such as a reduction in cigarettes smoked, adopting a smoking ban in the household, and perceived benefits of smoking cessation.^23,28^ Most studies that assessed abstinence (both biochemically verified and self-reported) reported abstinence at multiple endpoints, with the shortest being 2 weeks^24^ and the longest assessed at 52 weeks follow-up.^29,30^ Except for one study^36^ that assessed outcomes 6 weeks after the participants had stopped using the CA, all other studies assessed the immediate response directly after use.

**Figure 2.**
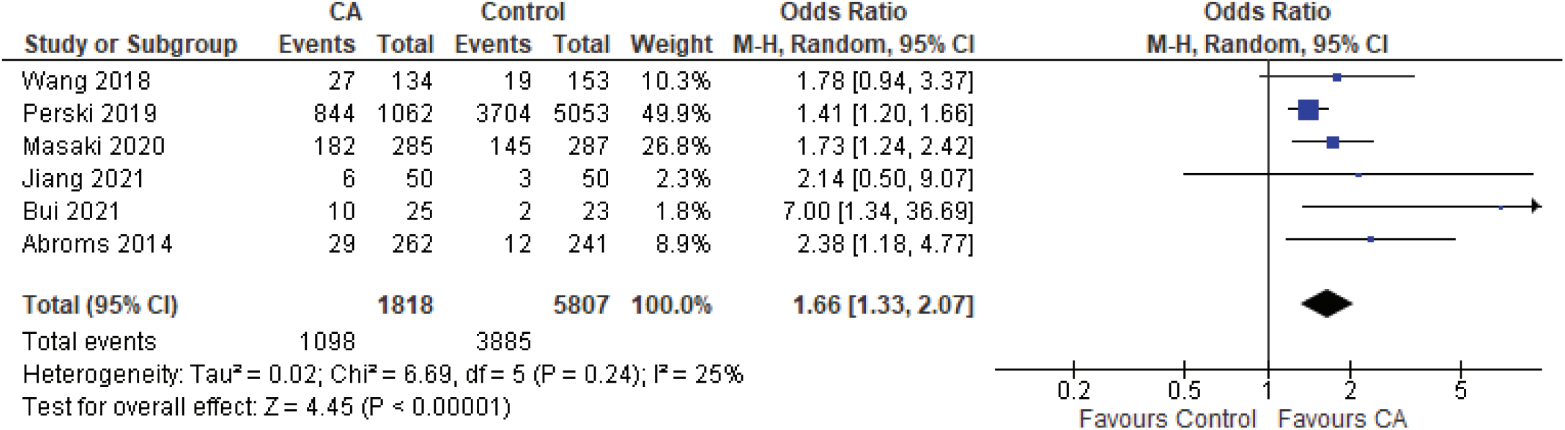
The effectiveness of conversational agent intervention for smoking cessation.

**Figure 3.**
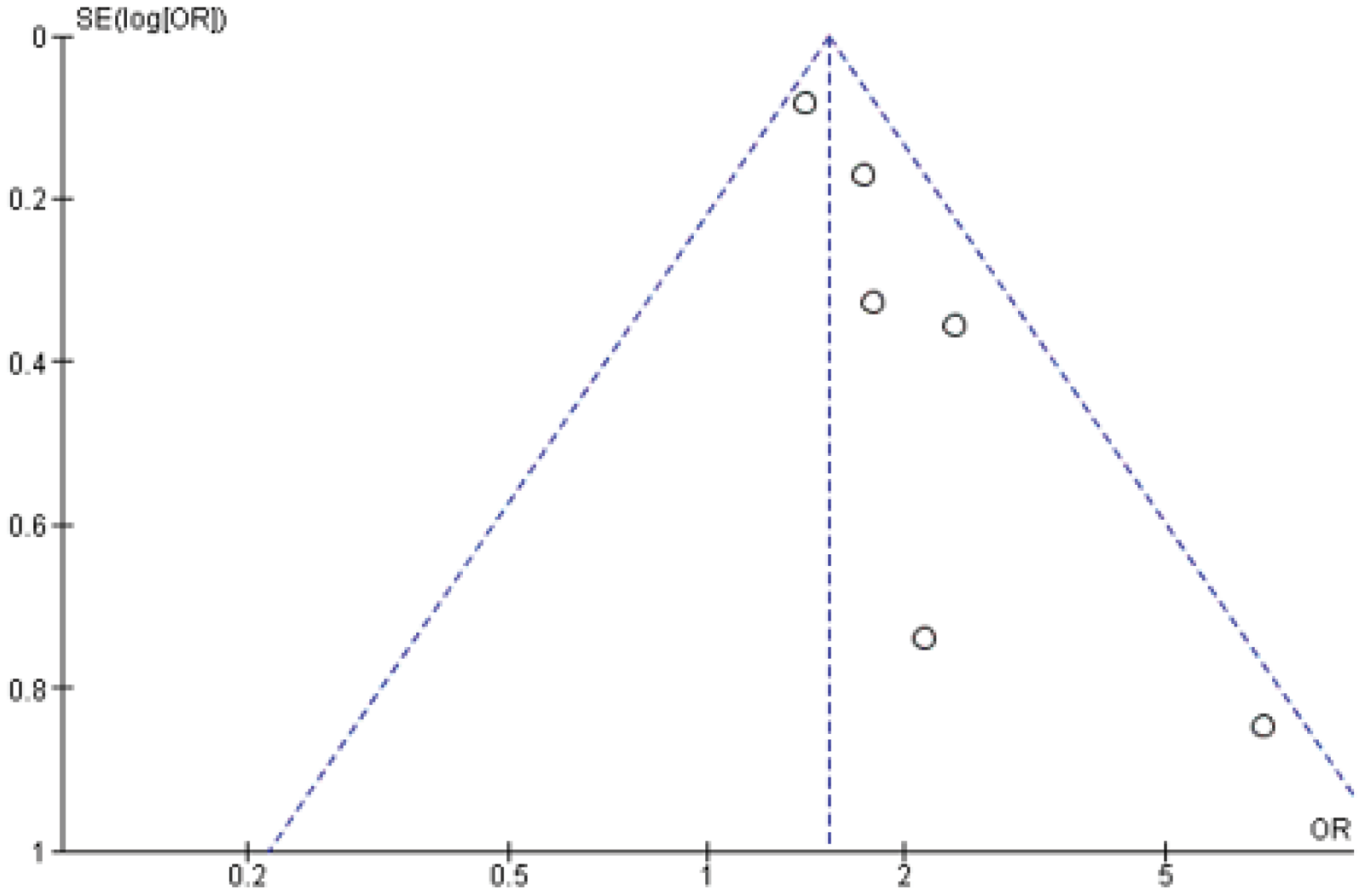
Funnel plot assessing publication bias.

A meta-analysis including six RCTs was performed to examine the overall effectiveness of the interventions (*n* = 7625). The study by White et al.^26^ was excluded since they compared the CA with an extra intervention element, and isolating the effect of CA intervention was therefore not possible. The sample-weighted odds ratio indicated that CA interventions significantly increased the odds of abstinence compared to control groups (odds ratio = 1.66, 95% CI = 1.33% to 2.07%, *p* < .001) (see Figure 2). Heterogeneity statistics indicated low heterogeneity (*I*^2^ = 25%) and no subgroup analysis was performed. Examination of the funnel plot indicated some asymmetry suggesting possible publication bias where unpublished studies with negative or smaller effects might be missing (see Figure 3).

We performed sensitivity analyses by iteratively removing studies that used only self-reported measures,^27,32^ with a high risk of bias,^32^ and with a considerably large sample weight.^27^ The results can be found in Supplementary File 4. In summary, removing the studies had no impact on the overall effects of the interventions, although heterogeneity slightly increased to 38% when the study with a high risk of bias was removed.

The remaining non-RCTs with pre-post designs also reported positive results regarding the interventions. Four of the non-RCTs reported abstinence with at least a 28.6% abstinence rate at follow-ups, compared to baseline.^24,29,31,35^ Moreover, 100% of participants (*n* = 23) in the study of Abroms et al.^24^ and 50% of participants (*n* = 3) in the study of Abdullah et al.^23^ reported having made at least one quit attempt after the intervention.

### Acceptability Evaluation

Generally, from studies that reported user experience outcomes (*k* = 10), participants reported high acceptability of the CA interventions. The majority of studies reported positive user experiences using a number of self-reported metrics. For example, participants reported that they were generally satisfied with the CA interventions,^23,33^ that they liked the agents or the interventions,^24,26,29^ that they felt comfortable interacting with the agents,^34^ that they found the intervention useful^24^ and easy to use,^34^ and that they would recommend the systems to other people.^24,34^ In addition to standard questionnaires, three studies that asked for open-ended user feedback also revealed a few challenges and barriers in using CA.^23,28,33^ The most commonly mentioned barriers included lack of human-likeness, lack of response coherence, limitations in the agents’ understanding and responding abilities, and inappropriate responses that frustrated users possibly due to the pre-scripted nature. Some participants in the study by Abdullah et al.^23^ mentioned that they would like more self-paced interaction, for example, being able to start the interaction as needed and not being pushed by the agent to set a quit date. Participants in the study by Jiang et al.^33^ expressed a strong interest in a more interactive approach with more freedom in texting in natural language. Last, some participants suggested extending the interaction duration.^23,33^

Studies reported on engagement with the CA interventions using a number of different metrics, and reporting was inconsistent. Most studies operationalized and measured engagement as the amount of usage, and they found overall high engagement. For example, studies reported that over 85% of participants sent at least one message to the agent,^25^ that 75% of participants read all messages,^24^ that participants interacted with the agent for 36 out 42 days,^33^ and that the agents increased the frequency of logins^27^ and increased the discussion in the chat.^32^

## Discussion

### Principal Findings

CAs are becoming increasingly implemented in health promotion as a promising tool to aid public health services. This review is the first to assess the effectiveness and acceptability of CAs in promoting smoking cessation. The CA interventions reviewed in this study varied considerably regarding system and intervention features, participants characteristics, comparison conditions, and cessation outcomes. Despite the heterogeneity in study designs and outcome evaluation methods used, all studies reported positive effects on smoking cessation-related outcomes, ranging from biochemically verified abstinence to self-reported perceived benefits of cessation. In addition, a meta-analysis of six RCTs demonstrated significantly higher odds of cessation when a CA was involved compared to control groups. This finding provides initial promise for the potential efficacy of CAs in promoting smoking cessation. However, an indication of potential publication bias was observed. Further, there was considerable variation in measurements and reporting of outcomes, which together indicate that the observed positive effects should be interpreted with caution. For example, some studies measured self-reported reduction in cigarette consumption after the intervention,^23^ whereas others assessed whether participants made any quit attempts during the intervention.^24^ One study did not measure cessation directly but asked for self-reported benefits of cessation, to indicate the potential effectiveness of the CA.^28^ Such inconsistencies in evaluation methods have been observed in previous systematic reviews on the use of CAs in other health-related areas.^38,39^ Notably, the majority of the studies did not preregister their study designs and analysis plans (except for Refs ^26^ and ^27^), which reduces the replicability and reproducibility of the results.^40^ Moreover, among the RCTs, less than half of the studies specifically mentioned the adherence to the intention-to-treat principle or the use of the complete sample set,^26,30,34^ which might lead to overestimation of the actual effect and potentially explained the observed publication bias.^41^ In conclusion, the present review found initial promise for the use of CA in assisting smoking cessation, but the actual effects need to be future examined, using more rigorous preregistered research, using standardized outcomes and analysis approaches.

Regarding the user experience of the CAs, results were mixed but a majority reported positive experiences, which illustrates initial user acceptance of the agents as the intervention vehicle. Interestingly, participants valued some aspects of the agents that are usually seen as unique in human–human counseling. For example, participants appreciated that the CA was supportive in interpersonal relationships and that they connected with the CA on a personal level.^23^ This is consistent with research demonstrating that people respond in social ways to computers as if they would respond to humans.^42^ Users’ appreciation of the relational aspect (as unique in human–human communication) is essential for long-term engagement, which predicts sustained smoking cessation.^38,43^ People often need several quit attempts to achieve abstinence, and long-term support is valued by smokers in the quitting process.^44,45^ However, the majority of the CAs in this review did not specify designs for long-term engagement, and the coincidence finding of users appreciating the relational aspects calls for more attention in future designs to build up the overall positive user experience observed in this review.

There were also a number of other limitations of the agents noted in this review. In addition to the aforementioned lack of relational aspects that hinder long-term engagement, CAs in the included papers and for healthcare oftentimes use pre-scripted utterances and only allow constrained user input to ensure controllability and avoid unwanted harm,^46,47^ while user experience might be compromised in the long term, because of the somewhat predictable nature of scripted interactions. In addition to the limitations in the CAs’ technical capabilities, participants expressed frustration when the agents gave them too much pressure to name a quit date^23^ or when the agents asked for self-reflection which participants found uncomfortable to answer.^28^ This echoes the joint research agenda in human-computer interaction and addiction counseling that focuses on the core relational factors such as being empathic and understanding and adapting to patients’ motivational states instead of taking a confrontational and demanding approach.^48,49^ Lack of control was another important barrier mentioned by participants, especially in the pace and length of the interaction. Participants prefer interacting with the agents at their own pace and in their own words (ie, more free texts rather than predefined keyword options). This is consistent with previous reviews on the use of CA interventions demonstrating that autonomy is a key factor in the experience with the agents and in ultimate behavioral change.^7^ To summarize, CAs appear to be an overall acceptable format for smoking cessation interventions, with areas for improvement suggested for future development.

### Future Directions

Theoretical frameworks for designing the contents of the chatbot intervention are essential to understanding the potential mechanism of behavioral change. However, theoretical frameworks were not reported in many of the studies included in this review. The lack of a theoretical foundation for chatbot intervention development has been noted in previous research.^38^ Among the reviewed studies that reported on theoretical frameworks, Social Cognitive Theory was the most frequently used framework.^24,25,33^ In addition, we found that the majority of behavioral change techniques were designed to increase persuasion (eg providing contingent rewards and facilitating goal setting),^27^ while relational strategies received less attention. Relational communicative strategies are being increasingly implemented in recent CA development, especially for mental health,^48^ and resulted in positive effects.^50^ However, current CAs for smoking cessation seem to focus less on the emotional and relational needs of the patients, while the patient–counselor relationship is essential to achieve long-term goals.^51^ Nonetheless, two studies in this review cited frameworks that emphasize stages of change,^28,34^ such as the Transtheoretical Model and Motivational Interviewing, which echoes participants’ need for empathic, non-demanding, and self-paced interventions that emerged from the qualitative feedback. Future studies could benefit from considering the literature on counseling and consulting and on human-computer interactions to develop CAs that can build and maintain a positive relationship with the users. For example, Bickmore et al.^48,52^ outlined a list of key theoretical considerations in designing relational agents for clinical purposes, such as therapeutic alliance and empathy. It is also encouraged to report on the theoretical foundation and behavioral change techniques to help ascertain the active ingredient of the interventions.

Engagement with the CAs was not reported consistently and the measures varied across studies, limiting the systematic assessment of the interventions. For example, several studies adopted the average length of interaction as an indicator of engagement,^24,25^ while some used frequency of interaction or logins to measure engagement.^26,27,33^ The number of user-initiated interactions was also used to assess user engagement.^25,29,33^ Previous reviews on the use of healthcare artificial intelligence systems also noted the inconsistency in measuring and reporting user engagement.^7,38^ Such inconsistencies could be problematic as it hinders the comparison across studies, while systematic summarization is essential given the infancy of this research area.^38^ Therefore, standardization of conceptualization and measures of user engagement is needed in future research. For example, Perski et al.^53^ conceptualized user engagement with digital behavioral change interventions in terms of both subjective experience and objective usage behaviors and suggested both scale questionnaires and standardized usage indicators as viable metrics for user engagement.

Similarly, the technical infrastructure was not reported in the majority of the studies, while technology can have a great impact on how users experience the intervention. With regard to chatbot dialogs, only three studies mentioned the dialogue management system and they were all rule-based.^28,32,35^ The utterances were human-authored and were sent to the users based on pre-defined rules. Rule-based agents are the majority of the current healthcare systems to ensure safety and controllability in sensitive domains.^54^ In terms of user input, most studies allowed a combination of fixed keywords and free texts, even though some participants mentioned that the agents’ ability to understand free texts was limited. In summary, the CAs in the reviewed studies seem to be largely constrained, thus ensuring the controllability and consistency in the delivery of content.^55^ However, they are less able to adapt to individual conversations and are, therefore, perceived as less natural and less engaging, which may hinder long-term interactions with users.^38,55^ Future research is encouraged to extend the initial CA effectiveness observed in this review, by improving the flexibility of the natural language interactions (moving away from pre-scripted interactions), while at the same time balancing this with controllability (making sure that interactions are constrained and do not go off the rails). Furthermore, various modalities (eg, embodied or non-embodied, speech- or text-based) of CA have been used for other health domains such as mental health and physical activities,^47^ while the agents identified in this review were all text-based. It will be important for future studies to compare different modalities and provide further insights into what works and for whom. Technical characteristics can have a great impact on users’ perception of and experience with the agents, and ultimately influence the intervention outcomes.^46,56^ Future studies are encouraged to provide descriptions of the technical infrastructure of the agents to allow a better understanding of the relationship between technology and health outcomes.

In summary, there is initial support for the use of CAs in smoking cessation, although no strong conclusions can be drawn yet, given indications of publication bias, lack of preregistered studies, and standardized outcomes. To better realize their potential and to have a more robust evaluation, we call for future research to further examine the long-term effect using more rigorous and standardized approaches.

On a broader note, CAs are being increasingly used in healthcare in general, with many products being developed and implemented over the decade showing positive effects in various behavior domains.^12,57^ Many of the findings reported in this paper generalize to CAs for stimulating other healthy behaviors. While recent advancement has enabled CAs to simulate human-like interactions, we would like to note that they should be considered as a supplementary tool rather than a replacement of human healthcare providers. CAs are useful as an on-demand addition when users need support and healthcare professionals only have limited availability. However, it is not yet clear whether the current generation of CAs lives up to its potential due to the great variation in their designs and evaluation. To ensure that they provide responsible and accountable support, further developments are needed to foster long-term engagement and understand the user-CA interactions. Moreover, it should be noted that most of the reviewed studies were conducted in developed countries and did not investigate sociodemographic factors in people’s experience with CAs. Recent rapid advances in technology can unintentionally increase health disparities^58^ by overlooking certain characteristics, needs, or preferences of potential users. To better realize the promise of the CAs and reduce the disparities in the use of such technology, it is crucial that future research include more diverse populations and consider disadvantaged groups in the process of CA development.

## Conclusion

Despite the limited body of evidence, this review provides initial promise for the effectiveness and acceptability of CAs for smoking cessation. Interventions using a CA reported better cessation outcomes compared with control conditions. User experience appeared to be overall positive, while special needs for relational components and self-paced interaction also emerged. The potential of such agents in assisting healthcare resulted in a rapid increase in both industrial development and publications, while standard measures and evaluations of such agents are still lacking, impeding the generalizability of the results. Additionally, theoretical and technical foundations of the agents need more attention in future development. Overall, the present findings demonstrate the potential of CAs for smoking cessation and suggest a clear need for further rigorous design and evaluation of such agents.

## Supplementary Material

A Contributorship Form detailing each author’s specific involvement with this content, as well as any supplementary data, are available online at https://academic.oup.com/ntr.

ntac281_suppl_Supplementary_File_S1

ntac281_suppl_Supplementary_File_S2

ntac281_suppl_Supplementary_File_S3

ntac281_suppl_Supplementary_File_S4

## Data Availability

The full search strategy can be found in supplementary materials. The full list of screened articles can be obtained from the corresponding author upon request.
