# Supplementary material for: Effectiveness and Acceptability of Conversational Agents for Smoking Cessation: A Systematic Review and Meta-analysis
Source: Nicotine Tob Res. 2022 Dec 12;25(7):1241–50. doi: 10.1093/ntr/ntac281 (PMC10256885; doi:10.1093/ntr/ntac281)
Supplement: ntac281_suppl_Supplementary_File_S1 [file ntac281_suppl_supplementary_file_s1.docx]

**Supplementary file 1.**

**PsycInfo (EBSCOhost)**

**#1 conversational agents**

AB ( chatbot OR "artificial intelligence" OR "conversation* agent" OR "dialogue system" OR "automated messag*" OR bot OR "chat agent" OR "relational agent" OR "virtual agent" OR "virtual assistan*" OR "virtual coach" OR "digital agent" OR "digital assistan*" OR "digital coach" ) OR TI ( chatbot OR "artificial intelligence" OR "conversation* agent" OR "dialogue system" OR "automated messag*" OR bot OR "chat agent" OR "relational agent" OR "virtual agent" OR "virtual assistan*" OR "virtual coach" OR "digital agent" OR "digital assistan*" OR "digital coach" ) OR SU ( chatbot OR "artificial intelligence" OR "conversation* agent" OR "dialogue system" OR "automated messag*" OR bot OR "chat agent" OR "relational agent" OR "virtual agent" OR "virtual assistan*" OR "virtual coach" OR "digital agent" OR "digital assistan*" OR "digital coach" ) OR KW ( chatbot OR "artificial intelligence" OR "conversation* agent" OR "dialogue system" OR "automated messag*" OR bot OR "chat agent" OR "relational agent" OR "virtual agent" OR "virtual assistan*" OR "virtual coach" OR "digital agent" OR "digital assistan*" OR "digital coach" )

10,781 hits

**#2 smoking cessation**

AB ( smoking OR tobacco OR nicotine OR cigarette* OR smok* OR "smoking cessation" OR "tobacco control" OR "stop smoking" OR "quit* smoking" OR "smok* abstinence" OR "smok* prevention" OR "smok* reduction" OR "smok* free" OR "cigarette* reduction"  ) OR TI ( smoking OR tobacco OR nicotine OR cigarette* OR smok* OR "smoking cessation" OR "tobacco control" OR "stop smoking" OR "quit* smoking" OR "smok* abstinence" OR "smok* prevention" OR "smok* reduction" OR "smok* free" OR "cigarette* reduction" ) OR SU ( smoking OR tobacco OR nicotine OR cigarette* OR smok* OR "smoking cessation" OR "tobacco control" OR "stop smoking" OR "quit* smoking" OR "smok* abstinence" OR "smok* prevention" OR "smok* reduction" OR "smok* free" OR "cigarette* reduction" ) OR KW ( smoking OR tobacco OR nicotine OR cigarette* OR smok* OR "smoking cessation" OR "tobacco control" OR "stop smoking" OR "quit* smoking" OR "smok* abstinence" OR "smok* prevention" OR "smok* reduction" OR "smok* free" OR "cigarette* reduction" )

70,041 hits

**#1 AND #2 🡪** 33 hits

**Web of Science**

#1 conversational agent

(TOPIC=( chatbot OR "artificial intelligence" OR "conversation* agent" OR "dialogue system" OR "automated messag*" OR bot OR "chat agent" OR "relational agent" OR "virtual agent" OR "virtual assistan*" OR "virtual coach" OR "digital agent" OR "digital assistan*" OR "digital coach")) AND (LA==("ENGLISH"))

66,831 hits

#2 smoking cessation

(TOPIC=( smoking OR tobacco OR nicotine OR cigarette* OR smok* OR "smoking cessation" OR "tobacco control" OR "stop smoking" OR "quit* smoking" OR "smok* abstinence" OR "smok* prevention" OR "smok* reduction" OR "smok* free" OR "cigarette* reduction" )) AND (LA==("ENGLISH"))

445,210 hits

**#1 AND #2 🡪**236 hits

**ACM Digital Library**

Title:(chatbot OR "artificial intelligence" OR "conversation* agent" OR "dialogue system" OR "automated messag*" OR bot OR "chat agent" OR "relational agent" OR "virtual agent" OR "virtual assistan*" OR "virtual coach" OR "digital agent" OR "digital assistan*" OR "digital coach") OR Abstract:(chatbot OR "artificial intelligence" OR "conversation* agent" OR "dialogue system" OR "automated messag*" OR bot OR "chat agent" OR "relational agent" OR "virtual agent" OR "virtual assistan*" OR "virtual coach" OR "digital agent" OR "digital assistan*" OR "digital coach") OR Keyword:(chatbot OR "artificial intelligence" OR "conversation* agent" OR "dialogue system" OR "automated messag*" OR bot OR "chat agent" OR "relational agent" OR "virtual agent" OR "virtual assistan*" OR "virtual coach" OR "digital agent" OR "digital assistan*" OR "digital coach")

7,532 hits

Title:(smoking OR tobacco OR nicotine OR cigarette* OR smok* OR "smoking cessation" OR "tobacco control" OR "stop smoking" OR "quit* smoking" OR "smok* abstinence" OR "smok* prevention" OR "smok* reduction" OR "smok* free" OR "cigarette* reduction") OR Abstract:(smoking OR tobacco OR nicotine OR cigarette* OR smok* OR "smoking cessation" OR "tobacco control" OR "stop smoking" OR "quit* smoking" OR "smok* abstinence" OR "smok* prevention" OR "smok* reduction" OR "smok* free" OR "cigarette* reduction") OR Keyword:(smoking OR tobacco OR nicotine OR cigarette* OR smok* OR "smoking cessation" OR "tobacco control" OR "stop smoking" OR "quit* smoking" OR "smok* abstinence" OR "smok* prevention" OR "smok* reduction" OR "smok* free" OR "cigarette* reduction")

515 hits

#1 AND #2 🡪 9 hits

**IEEE Xplore**

#1 Conversational agent

“All Metadata”: chatbot OR "artificial intelligence" OR "conversation* agent" OR "dialogue system" OR "automated messag*" OR bot OR "chat agent" OR "relational agent" OR "virtual agent" OR "virtual assistan*" OR "virtual coach" OR "digital agent" OR "digital assistan*" OR "digital coach"

308,455 hits

#2 smoking cessation

“All Metadata”: smoking OR tobacco OR nicotine OR cigarette* OR smok* OR "smoking cessation" OR "tobacco control" OR "stop smoking" OR "quit smoking" OR “quitting smoking” OR "smok* abstinence" OR "smok* prevention" OR "smok* reduction" OR "smok* free" OR "cigarette* reduction"

4,783 hits

#1 AND #2 🡪 433 hits

**Medline (EBSCOhost)**

#1 conversational agents

AB (chatbot OR "artificial intelligence" OR "conversation* agent" OR "dialogue system" OR "automated messag*" OR bot OR "chat agent" OR "relational agent" OR "virtual agent" OR "virtual assistan*" OR "virtual coach" OR "digital agent" OR "digital assistan*" OR "digital coach") OR TI (chatbot OR "artificial intelligence" OR "conversation* agent" OR "dialogue system" OR "automated messag*" OR bot OR "chat agent" OR "relational agent" OR "virtual agent" OR "virtual assistan*" OR "virtual coach" OR "digital agent" OR "digital assistan*" OR "digital coach") OR SU ( chatbot OR "artificial intelligence" OR "conversation* agent" OR "dialogue system" OR "automated messag*" OR bot OR "chat agent" OR "relational agent" OR "virtual agent" OR "virtual assistan*" OR "virtual coach" OR "digital agent" OR "digital assistan*" OR "digital coach")

44,141 hits

#2 smoking cessation

AB ( smoking OR tobacco OR nicotine OR cigarette* OR smok* OR "smoking cessation" OR "tobacco control" OR "stop smoking" OR "quit* smoking" OR "smok* abstinence" OR "smok* prevention" OR "smok* reduction" OR "smok* free" OR "cigarette* reduction") OR TI ( smoking OR tobacco OR nicotine OR cigarette* OR smok* OR "smoking cessation" OR "tobacco control" OR "stop smoking" OR "quit* smoking" OR "smok* abstinence" OR "smok* prevention" OR "smok* reduction" OR "smok* free" OR "cigarette* reduction") OR SU (smoking OR tobacco OR nicotine OR cigarette* OR smok* OR "smoking cessation" OR "tobacco control" OR "stop smoking" OR "quit* smoking" OR "smok* abstinence" OR "smok* prevention" OR "smok* reduction" OR "smok* free" OR "cigarette* reduction" )

401,746 hits

#1 AND #2 🡪 213 hits

**EMBASE**

#1 conversational agent

chatbot:ti,ab,kw OR 'artificial intelligence':ti,ab,kw OR 'conversation* agent':ti,ab,kw OR 'dialogue system':ti,ab,kw OR 'automated messag*':ti,ab,kw OR bot:ti,ab,kw OR 'chat agent':ti,ab,kw OR 'relational agent':ti,ab,kw OR 'virtual agent':ti,ab,kw OR 'virtual assistan*':ti,ab,kw OR 'virtual coach':ti,ab,kw OR 'digital agent':ti,ab,kw OR 'digital assistan*':ti,ab,kw OR 'digital coach':ti,ab,kw

29,263 hits

#2 smoking cessation

smoking:ti,ab,kw OR tobacco:ti,ab,kw OR nicotine:ti,ab,kw OR cigarette*:ti,ab,kw OR smok*:ti,ab,kw OR 'smoking cessation':ti,ab,kw OR 'tobacco control':ti,ab,kw OR 'stop smoking':ti,ab,kw OR 'quit* smoking':ti,ab,kw OR 'smok* abstinence':ti,ab,kw OR 'smok* prevention':ti,ab,kw OR 'smok* reduction':ti,ab,kw OR 'smok* free':ti,ab,kw OR 'cigarette* reduction':ti,ab,kw

558,818 hits

#1 AND #2 🡪 248

**Communication and Mass Media Complete (EBSCOhost)**

#1 conversational agent

TI ( chatbot OR "artificial intelligence" OR "conversation* agent" OR "dialogue system" OR "automated messag*" OR bot OR "chat agent" OR "relational agent" OR "virtual agent" OR "virtual assistan*" OR "virtual coach" OR "digital agent" OR "digital assistan*" OR "digital coach" ) OR SU ( chatbot OR "artificial intelligence" OR "conversation* agent" OR "dialogue system" OR "automated messag*" OR bot OR "chat agent" OR "relational agent" OR "virtual agent" OR "virtual assistan*" OR "virtual coach" OR "digital agent" OR "digital assistan*" OR "digital coach" ) OR AB ( chatbot OR "artificial intelligence" OR "conversation* agent" OR "dialogue system" OR "automated messag*" OR bot OR "chat agent" OR "relational agent" OR "virtual agent" OR "virtual assistan*" OR "virtual coach" OR "digital agent" OR "digital assistan*" OR "digital coach" ) OR KW ( chatbot OR "artificial intelligence" OR "conversation* agent" OR "dialogue system" OR "automated messag*" OR bot OR "chat agent" OR "relational agent" OR "virtual agent" OR "virtual assistan*" OR "virtual coach" OR "digital agent" OR "digital assistan*" OR "digital coach" )

1,568 hits

#2 smoking cessation

TI ( smoking OR tobacco OR nicotine OR cigarette* OR smok* OR "smoking cessation" OR "tobacco control" OR "stop smoking" OR "quit* smoking" OR "smok* abstinence" OR "smok* prevention" OR "smok* reduction" OR "smok* free" OR "cigarette* reduction" ) OR SU ( smoking OR tobacco OR nicotine OR cigarette* OR smok* OR "smoking cessation" OR "tobacco control" OR "stop smoking" OR "quit* smoking" OR "smok* abstinence" OR "smok* prevention" OR "smok* reduction" OR "smok* free" OR "cigarette* reduction" ) OR AB ( smoking OR tobacco OR nicotine OR cigarette* OR smok* OR "smoking cessation" OR "tobacco control" OR "stop smoking" OR "quit* smoking" OR "smok* abstinence" OR "smok* prevention" OR "smok* reduction" OR "smok* free" OR "cigarette* reduction" ) OR KW ( smoking OR tobacco OR nicotine OR cigarette* OR smok* OR "smoking cessation" OR "tobacco control" OR "stop smoking" OR "quit* smoking" OR "smok* abstinence" OR "smok* prevention" OR "smok* reduction" OR "smok* free" OR "cigarette* reduction" )

3,298 hits

#1 AND #2 🡪 1 hit

**CINAHL Complete (EBSCOhost)**

#1 conversational agent

TI ( chatbot OR "artificial intelligence" OR "conversation* agent" OR "dialogue system" OR "automated messag*" OR bot OR "chat agent" OR "relational agent" OR "virtual agent" OR "virtual assistan*" OR "virtual coach" OR "digital agent" OR "digital assistan*" OR "digital coach") OR SU ( chatbot OR "artificial intelligence" OR "conversation* agent" OR "dialogue system" OR "automated messag*" OR bot OR "chat agent" OR "relational agent" OR "virtual agent" OR "virtual assistan*" OR "virtual coach" OR "digital agent" OR "digital assistan*" OR "digital coach") OR AB chatbot OR "artificial intelligence" OR "conversation* agent" OR "dialogue system" OR "automated messag*" OR bot OR "chat agent" OR "relational agent" OR "virtual agent" OR "virtual assistan*" OR "virtual coach" OR "digital agent" OR "digital assistan*" OR "digital coach")

10,234 hits

#2 smoking cessation

TI ( smoking OR tobacco OR nicotine OR cigarette* OR smok* OR "smoking cessation" OR "tobacco control" OR "stop smoking" OR "quit* smoking" OR "smok* abstinence" OR "smok* prevention" OR "smok* reduction" OR "smok* free" OR "cigarette* reduction" ) OR SU ( smoking OR tobacco OR nicotine OR cigarette* OR smok* OR "smoking cessation" OR "tobacco control" OR "stop smoking" OR "quit* smoking" OR "smok* abstinence" OR "smok* prevention" OR "smok* reduction" OR "smok* free" OR "cigarette* reduction" ) OR AB ( smoking OR tobacco OR nicotine OR cigarette* OR smok* OR "smoking cessation" OR "tobacco control" OR "stop smoking" OR "quit* smoking" OR "smok* abstinence" OR "smok* prevention" OR "smok* reduction" OR "smok* free" OR "cigarette* reduction" )

147,524 hits

#1 AND #2 🡪 72 hits
