# Supplementary material for: Effectiveness and Acceptability of Conversational Agents for Smoking Cessation: A Systematic Review and Meta-analysis
Source: Nicotine Tob Res. 2022 Dec 12;25(7):1241–50. doi: 10.1093/ntr/ntac281 (PMC10256885; doi:10.1093/ntr/ntac281)
Supplement: ntac281_suppl_Supplementary_File_S4 [file ntac281_suppl_supplementary_file_s4.docx]

**Supplementary file 4.**

Sensitivity Analyses

Removing studies that used only self-reported abstinence:

**
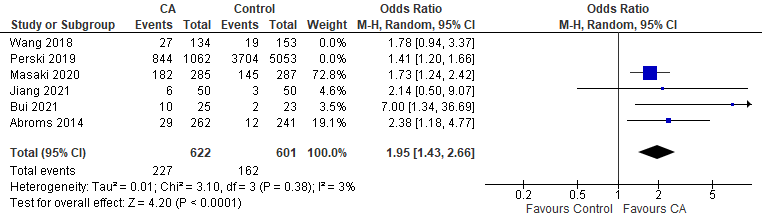
**

Removing studies that have a high risk of bias:

**
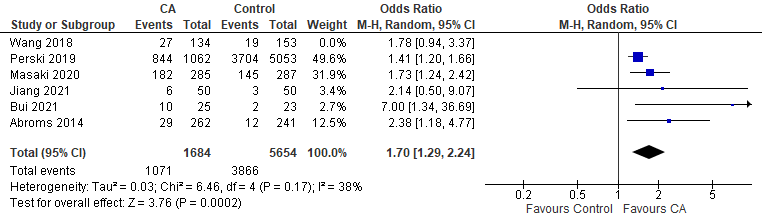
**

Removing studies that have a large sample weight:

**
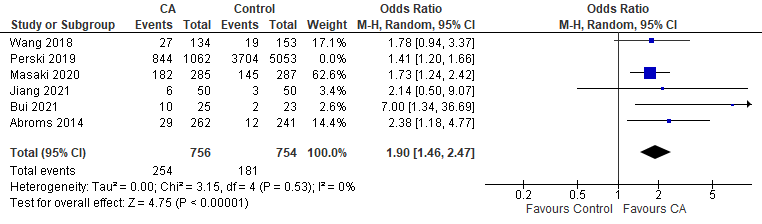
**
